# Expression of Concern: ChainAgile: A framework for the improvement of Scrum Agile distributed software development based on blockchain

**DOI:** 10.1371/journal.pone.0331232

**Published:** 2025-09-02

**Authors:** The PLOS One Editors

After this article [1] was published, the *PLOS One* Editors identified concerns about the article’s peer review. In addition, there are concerns over citation patterns in the article, including:

- Numerous statements in the Introduction that are not supported by data, or by published and cited work.
- The article cited as Reference 15 was retracted after [1] was published due to concerns about potential manipulation of the publication process.

We regret that the issues were not addressed prior to the article’s publication.

The first author responded to the above concerns, but the Editors do not consider the provided information to be sufficient to resolve the concerns.

In light of the cumulative issues, the *PLOS One* Editors issue this Expression of Concern. Readers are advised to interpret the article [1] with caution.
